# Controlled sol-gel nanoassembly of Au_25_(SR)_18_ nanoclusters for structural and functional integration

**DOI:** 10.3389/fchem.2026.1982602

**Published:** 2026-10-12

**Authors:** Mahdi Hesari, Marysa Colvin

**Affiliations:** Department of Chemistry, State University of New York at Oswego, Oswego, NY, United States

**Keywords:** 3-mercaptopropyl(trimethoxysilane), Au_25_(SR)_18_, nanoassembly, reductive catalysis, sol-gel

## Abstract

Structural assembly of nanomaterials imparts new and enhanced properties to individual components. The “Sol-gel” approach has been a traditional technique to connect diverse types of materials. Here, 3-mercaptopropyl (trimethoxysilane) (MPTS), a dual-functional ligand, has been employed to assemble one of the popular members of atomically precise nanoclusters–Au_25_^−^ and connect individual nanoclusters via the formation of the Si(OR)_4_ “sol-gel” network during the purification process. Depending on the purification procedure, a particle or film of Au_25_-SiO_2_ was achieved and characterized using different techniques. The obtained superstructures of nanomaterials were utilized as a catalyst for the nitrophenol hydrogenation reaction, revealing a 2.5 times faster reaction rate of Au_25_-SiO_2_-film relative to the particle.

## 1 Introduction

Superstructures are formed from self-assembled building blocks exhibiting collective optical, electronic, and magnetic properties (Huang et al., 2001; Erb et al., 2009; Levchenko et al., 2017; Freeman et al., 2018; Girard et al., 2019; Wu et al., 2019; Zhang Z. et al., 2022). Their properties and performance go beyond simply mixing the original components (Gao and Tang, 2011; Maity et al., 2025; Huang et al., 2026). The uniformity of the building blocks in superstructures is crucial for their application, enabling precise control over their combined properties (Li et al., 2022; Sen and Basu, 2026).

**SCHEME 1:**
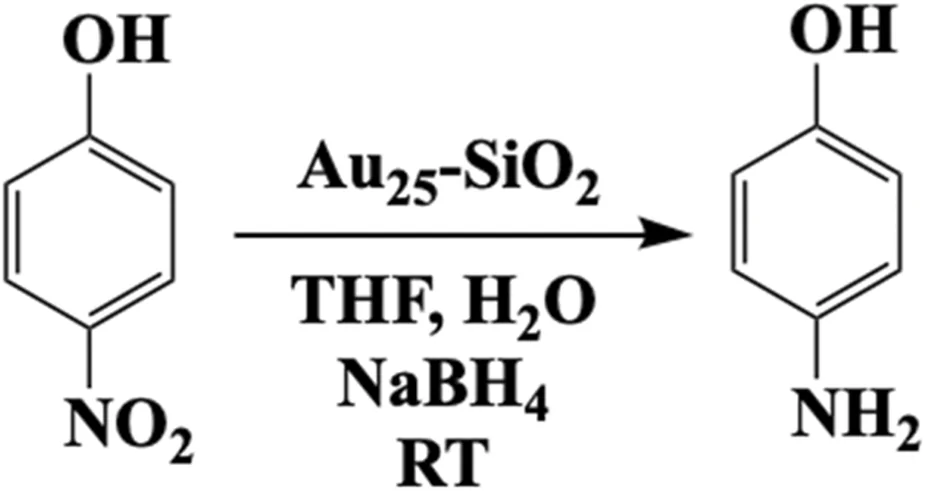
Schematic of the catalytic *p*-nitrophenol to aminophenol reaction.

Nanocluster chemistry has garnered significant interest recently due to its unique structures (Li and Jin, 2020) and properties (Jin et al., 2016; Kang and Zhu, 2020; Mitchell and Pérez-Ramírez, 2021; He et al., 2026), which can be exploited for various applications in energy (Hesari and Ding, 2017; Kawawaki et al., 2021a), catalysis (Yamazoe et al., 2014; Kawawaki et al., 2021b; Fan et al., 2024; Liu M. et al., 2024; Liu Y. et al., 2024), optics (Yu et al., 2019; Sun et al., 2024; Guo et al., 2026; Mazumder et al., 2026), bioimaging (Zhang C. et al., 2022; Liu F. et al., 2024), biohybrid systems (Yang et al., 2022; Okoro et al., 2023), and sensor design (Kumar et al., 2011; Kwak et al., 2014). There are several essential parameters, such as noble metal (Fields-Zinna et al., 2009; Kumara and Dass, 2011; Kuai et al., 2012; Kumara and Dass, 2012; Negishi et al., 2012; Gottlieb et al., 2013; Kauffman et al., 2013), protecting ligands (Schaaff and Whetten, 2000; Negishi et al., 2005; Jung et al., 2012; Kumar and Jin, 2012), and synthesis solvent (Liu et al., 2013), which alter the nanocluster’s size and physical and chemical properties (Negishi et al., 2013). Traditionally, the Brust-Schiffrin method, or two-phase method, has been employed to prepare a wide range of noble metal nanoclusters (Brust et al., 1994). Recently, a one-pot synthesis procedure has been developed to produce gold clusters of different sizes with higher monodispersity (Wu et al., 2009). Utilizing these synthetic procedures and alternating ingredient compositions results in a combination of a wide range of, for example, gold clusters with the general formula of Au_*x*_ (SR)_*y*_, where (*x*, *y*)– for example–are (20,16) (Zhu et al., 2009), (23,16) (Das et al., 2013; Hesari and Workentin, 2014), (24,16) (Zhu et al., 2010a) (25,18), (Wu et al., 2009) (28,20), (Zeng et al., 2013), (30,18) (Crasto and Dass, 2013), (36,24) (Nimmala and Dass, 2011), (38,24) (Toikkanen et al., 2008), (40,24) (Qian et al., 2010), (67,35) (Nimmala et al., 2013), (102,44) (Jadzinsky et al., 2007), and (144,60) (Qian and Jin, 2009; Parker et al., 2010a). Among a diverse range of noble metal nanoclusters, Au_25_(SR)_18_ nanoclusters have found numerous applications in catalysis (Zhu et al., 2010a), electrocatalysis (Kauffman et al., 2012), electrogenerated chemiluminescence (ECL) (Hesari et al., 2014a; Hesari et al., 2014b; Hesari and Ding, 2021a; Hesari and Ding, 2021b), and photoluminescence (Wang et al., 2012; Wu et al., 2012) due to their extraordinary optical, electrochemical (Parker et al., 2010b), and catalytic (Zhu et al., 2010b) properties. The preparation methods used to make those small gold nanoclusters with various protecting ligands mainly include thiols (HS-R). Importantly, the residual (–R) group can impart distinct properties to the resulting nanoclusters, such as water solubility (Negishi et al., 2005; Wu et al., 2009; Kumar and Jin, 2012).

Using a ligand that can anchor nanoclusters is important, as it enables fabrication of nanoclusters on various surfaces and their use as building blocks. Furthermore, in catalysis studies that typically occur at high temperatures under highly oxidizing or reducing conditions, it is crucial to retain the small size of nanoclusters for as long as possible. For example, Kraos and colleagues decorated gold nanoparticles with a silica (SiO_2_) layer using a phase exchange approach (Schulzendorf et al., 2010). Alternatively, Weng et al. (2018) decorated the surface of SiO_2_ particles with Au(SG) nanoclusters, which were subsequently loaded onto TiO_2_ particles to yield a stable photoactive catalyst. 3-mercaptopropyl (trimethoxysilane) (MPTS) is a widely used “sol-gel precursor” (Mirzaei et al., 2013) to stabilize quantum-sized Au nanoclusters. MPTS is a dual-function thiol ligand, which can engage with Au^3+^ and form a typical Au-S covalent bond. The presence of a trimethoxysilane group is a known sol-gel agent, which undergoes hydrolysis and condensation (Karaca, 2021) and assists with surface reinforcement, which is important in heterogeneous catalysis. The resulting Au nanoclusters were encapsulated in a transparent silica-based gel that stabilizes them in harsh solutions and at high temperatures. In addition, all the unique optical features of these nanoclusters are retained upon gel formation (Samanta et al., 2012). This type of silica-protected metal nanocluster has been synthesized through various preparation protocols (Guerrero-Martínez et al., 2010). These protocols include multistep procedures in which the silane-thiolated ligand is introduced into the precursors, followed by heating in acidic or basic solutions (Qi et al., 2008; Habeeb Muhammed and Pradeep, 2011). This procedure involved embedding gold cores within the silica shell. In most cases, MPTS has been used. Gao and colleagues encapsulated Au_25_ nanoclusters in SiO_2_ particles using a two-step protocol (Chen et al., 2019). The Au_25_@SiO_2_ obtained was further used to convert acetylene to ethylene, yielding 98% at 280 °C. In all the mentioned studies, the shape of the embedded Au_25_@SiO_2_ was not discussed, whereas this parameter could play a crucial role in the catalytic activity of materials. Here, we explored whether the form and thickness of the SiO_2_ layer influence the catalytic performance.

Herein, we report the one-pot synthesis of Au_25_ clusters protected with MPTS, which can be embedded in a silica shell film or particle, depending on the purification procedure (Experimental details are provided in the Supplementary Material). The final product in both thin film and grain forms was characterized by UV-Visible and photoluminescence spectroscopy, which confirmed the optical features as previously reported for Au_25_(SR)_18_ nanoclusters. Additionally, scanning electron microscopy (SEM), energy-dispersive X-ray spectroscopy (EDX), and X-ray photoelectron spectroscopy (XPS) provided information on the compositional details of the purified, SiO_2_-embedded Au_25_. X-ray powder diffraction (XRD) of the products exhibits features similar to those reported for Au_25_(SR)_18_, supporting the proposed structure.

## 2 Materials and methods

### 2.1 Materials

Tetrachloroaurate trihydrate, sodium borohydride, tetrahydrofuran, tetra-*n*-octyl ammonium bromide, and 3-mercaptopropyl (trimethoxysilane) (MPTS) were purchased from Aldrich. Sephadex®LH-20 was also purchased from Sigma-Aldrich.

### 2.2 Instrument

UV-visible spectra were recorded on a Thermo Evolution 220 UV using a 1 cm quartz cuvette in THF as a reference solvent in the range of 300–900 nm. Solid-state UV-visible measurements were conducted using a Carry 5000 spectrometer. The X-ray photoelectron spectroscopy (XPS) analyses were carried out with a Kratos AXIS Ultra spectrometer using a monochromatic Al K(alpha) source (15 mA, 14 kV). XPS can detect all elements except hydrogen and helium, probes the surface of the sample to a depth of 5–7 nm, and has detection limits ranging from 0.1 to 0.5 atomic percent depending on the element. The instrument work function was calibrated to give a binding energy (BE) of 83.96 eV for the Au 4f7/2 line for metallic gold, and the spectrometer dispersion was adjusted to give a BE of 932.62 eV for the Cu 2p3/2 line of metallic copper. The Kratos charge neutralizer system was used on all specimens. Samples were mounted on a non-conductive double-sided adhesive tape. Survey scan analyses were carried out with an analysis area of 300 × 700 microns and a pass energy of 160 eV. High-resolution analyses were carried out with an analysis area of 300 x 700 microns and a pass energy of 20 eV. Spectra have been charge-corrected to the main line of the carbon 1s spectrum (adventitious carbon) set to 284.8 eV. Spectra were analyzed using Casa XPS software (version 2.3.14). The S 2p spectra are fitted with pairs of peaks that model the spin-orbit doublets present (S 2p3/2 and S 2p1/2). Each doublet pair is constrained to have peaks of equal full-width at half maximum (FWHM), a spacing of 1.18 eV, and an area ratio of 2:1. The Cl 2p and Br 3d spectra are also fitted with a suitable spin-orbit doublet structure. Powder X-ray diffraction of the sample was conducted on a Rigaku RTP 300 using Co Kα radiation (λ = 1.790,260).

SEM images were recorded on a JEOL JSM-6610LV Scanning Electron Microscope with an EDX instrument. The same instrument was used to obtain EDX elemental analysis, in which the beam energy was set at 10 keV, and sample measurements were done within 600 s.

The photoluminescence spectrum was collected using a BRDD CCD camera (Andor iDus 401) connected to a Kymra i193 spectrograph. A 532 nm solid-state laser was later used as an excitation source. The PL spectra in both solution and solid-state were accumulated for 20 s.

A sub-sample of Au_25_(SC_2_H_4_Si(OCH3)_3_)_18_.TOA in THF was diluted 10 times using dry THF. Then, 10 µL of the solution was directly injected into a capillary of a microTOF mass spectrometer. The nebulizer was set at 1.0 Bar, and the capillary voltage was 4500 V. The spectrum was collected in positive ion polarity mode.

### 2.3 Synthesis of Au_25_(SC_2_H_4_Si(OCH_3_)_3_)_18_.TOA nanocluster

In a tri-neck 250 mL round-bottom flask, 1.28 mmol HAuCl_4_ · 3H_2_O and 1.35 mmol tetra-*n*-octylammonium bromide were dissolved in 50 mL THF. The solution was cooled down using an ice bath for 30 min. Then, 7.8 mmol of MPTS was added dropwise to the mixture. After 30 min, the solution color gradually turned to pale yellow. After 2 h, sodium borohydride (12.8 mmol) was dissolved in 10 mL of ice-cold water and added to the above solution rapidly under fast stirring. The solution color changed to dark brown, indicating nanocluster formation. The reaction was stirred for 24 h, and the formation of the nanocluster was monitored using UV-visible spectroscopy (Supplementary Figure S1). Then, the solution was gravity-filtered to separate unreacted Au(I)-S–intermediate polymers. The homogeneous bright brown solution was further used for the “sol-gel” treatments as described in the following sections.

### 2.4 Sol-gel approaches towards the synthesis of Au_25_–SiO_2_ network

Then, two approaches to treat the “sol-gel” system were taken as follows:

Procedure (A). (i.e., Au_25_-SiO_2_-particle) follows typical nanocluster purification, during which the crude product solvent is rotary-evaporated, leaving an oily residue. Next, the oily material was gradually purified with ethanol to remove the excess thiol. It has been observed that during the process, a solid phase (i.e., particles) forms due to the formation of the -(-Si-O-Si-) “sol-gel” network in the presence of residual water in the THF solution and added methanol.

Procedure (B). (i.e., Au_25_-SiO_2_-film) includes purifying the Au_25_(SC_2_H_4_Si(OCH_3_)_3_)_18_.TOA using the size exclusion chromatography (SEC) technique (Knoppe et al., 2011). Supplementary Figure S2 displays SEC during the purification process. Sephadex LH-20 was used for column chromatography in a typical separation, which allows for the separation of molecules based on their molecular weight. One may notice that a dark brown band is eluted by the solvent, THF, through the SEC column—the purified Au_25_(SC_2_H_4_Si(OCH_3_)_3_)_18_.TOA was collected, and the solvent was then removed by rotary evaporation at room temperature. Interestingly, due to (1) the removal of the excess thiol, (2) the absence of ethanol (and/or methanol), and (3) the low percentage of water in the solution, a thin film of Au_25_(SC_2_H_4_Si(OCH_3_)_3_)_18_.TOA embedded in SiO_2_ was formed.

## 3 Results and discussion

The UV-Visible spectrum of the Au_25_-SiO_2_ particle, a chunky dark brownish solid, was collected using solid-state UV-Visible spectroscopy. The obtained spectrum (Figure 1, black trace) reveals the Au_25_ nanocluster’s features at a wavelength higher than 550 nm. One can observe that the absorption valleys at 430 nm and 615 nm, a maximum at 680 nm, and a feature at 8ible features of Au_25_(SR)_18_^–^ at 400, 447, 556, and 685 nm (Wu et al., 2009; Parker et al., 2010b). The UV-visible spectrum of the Au_25_-SiO_2_-film also shows the same features as displayed in Figure 1, with the blue trace representing the identical spectral features of Au_25_.

**FIGURE 1:**
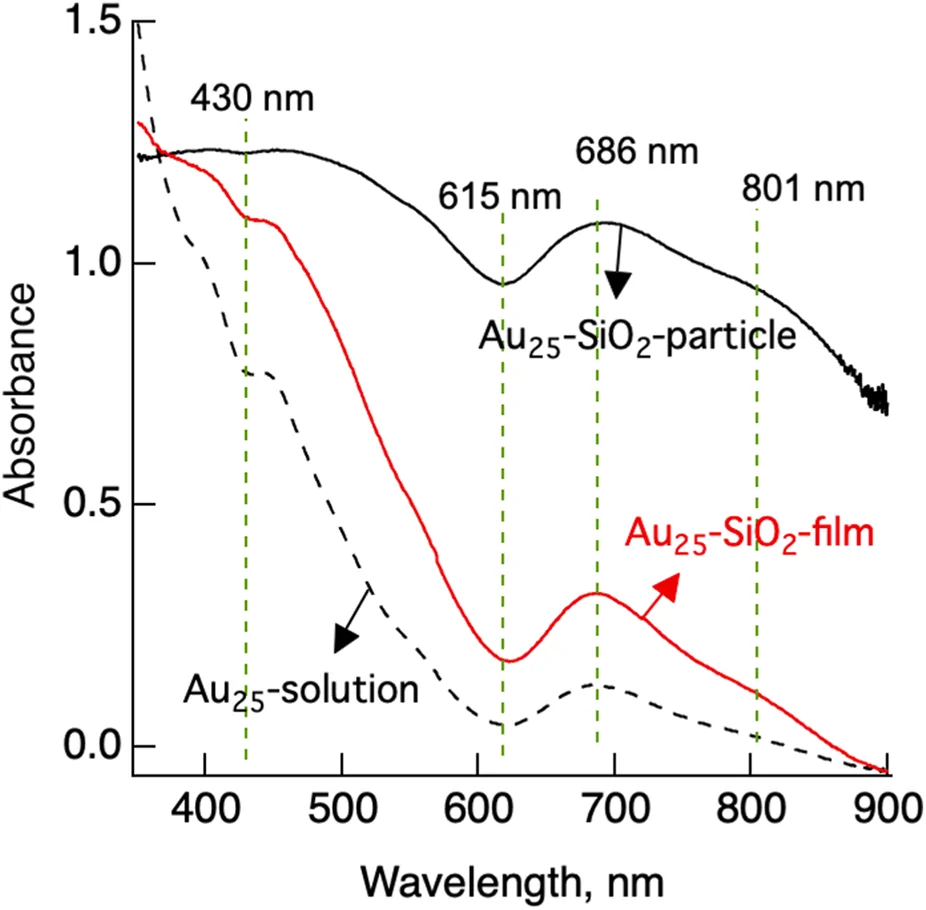
UV-visible spectroscopy of Au_25_(SC_2_H_4_Si(OCH_3_)_3_)_18_.TOA. **(A)** The solid-state spectrum of Au_25_-in-particle (the black trace), and **(B)** Au_25_-in-film (the blue dashed line). In both panels, the red trace represents Au_25_(SC_2_H_4_Si(OCH_3_)_3_)_18_.TOA in THF solution as a reference.

Before further characterizing Au_25_-SiO_2_-particle and Au_25_-SiO_2_-film, mass spectrometry of the Au_25_(SC_2_H_4_Si(OCH_3_)_3_)_18_.TOA in the THF solution was explored. A sub-sample of the Au_25_(SC_2_H_4_Si(OCH_3_)_3_)_18_.TOA in THF was diluted with dry THF and injected into a microTOF mass spectrometer. A diluted solution of the sample in dry THF was injected directly into the instrument. Due to the fragile structure of the used thiol (HS-C_2_H_4_-Si-(OCH_3_)_3_) Figure 2A displays the mass spectrum of Au_25_(SC_2_H_4_Si(OCH_3_)_3_)_18_.TOA. The obtained spectrum (Figure 2A) reveals a dominant mass region corresponding to small ionic and Au_13_ core fragments (m/z < 2,000 Da) (Angel et al., 2010). In addition, one can observe several peaks in a mass range of m/z∼2,000–3,000 Da that represent 3+ and 4+ charge states of Au_25_(SC_2_H_4_Si(OCH_3_)_3_)_18_.TOA–fragments with m/z values of 2700.3 and 2062.9 Da. Further analysis in this mass range revealed that the mass difference between 3+ and 4+ (*ca.* m/z: ∼547 Da) is related to the loss of 2×Au-SC_2_H_4_Si (m/z (Au-SC_2_H_4_Si): 285.2) (Figure 2B). A similar mass loss was observed in the mass range 2700.3–3200.7 Da. This observation supports the thiol’s fragile structure. More detailed fragmentation information was revealed when mass regions were further expanded. Figures 2C,D display the mass ranges 3,600–3,800 and 3,000–3,300 Da. One can identify several mass differences between observed peaks of Δm/z 45 Da, corresponding to 3 CH_3_ units. Figure 2E highlights the isotopic pattern of two adjacent–CH_3_ groups attached to a remaining fragment in the m/z range of 3,145 and 3,215 Da. This supports the surface ligand fragmentations. The Dass group also reports similar fragmentation behavior for Au_23_(SR)_16_ and Au_30_(SR)_18_ decorated with tert-butyl thiol (Wijesinghe et al., 2024): The–CH_3_ fragmentation in HS-C_2_H_4_-Si-(OCH_3_)_3_ would resemble the dissociation of–CH_3_ in HS-C(CH_3_)_3_, either in homolytic or heterolytic mechanisms. The loss of–CH_3_ groups was also observed in other m/z ranges, as depicted with double-headed arrows in Figures 2F,G. Our analysis revealed 51 –CH_3_ groups across different mass ranges, which matches the theoretical number of –CH_3_ groups, approximately 54.

**FIGURE 2:**
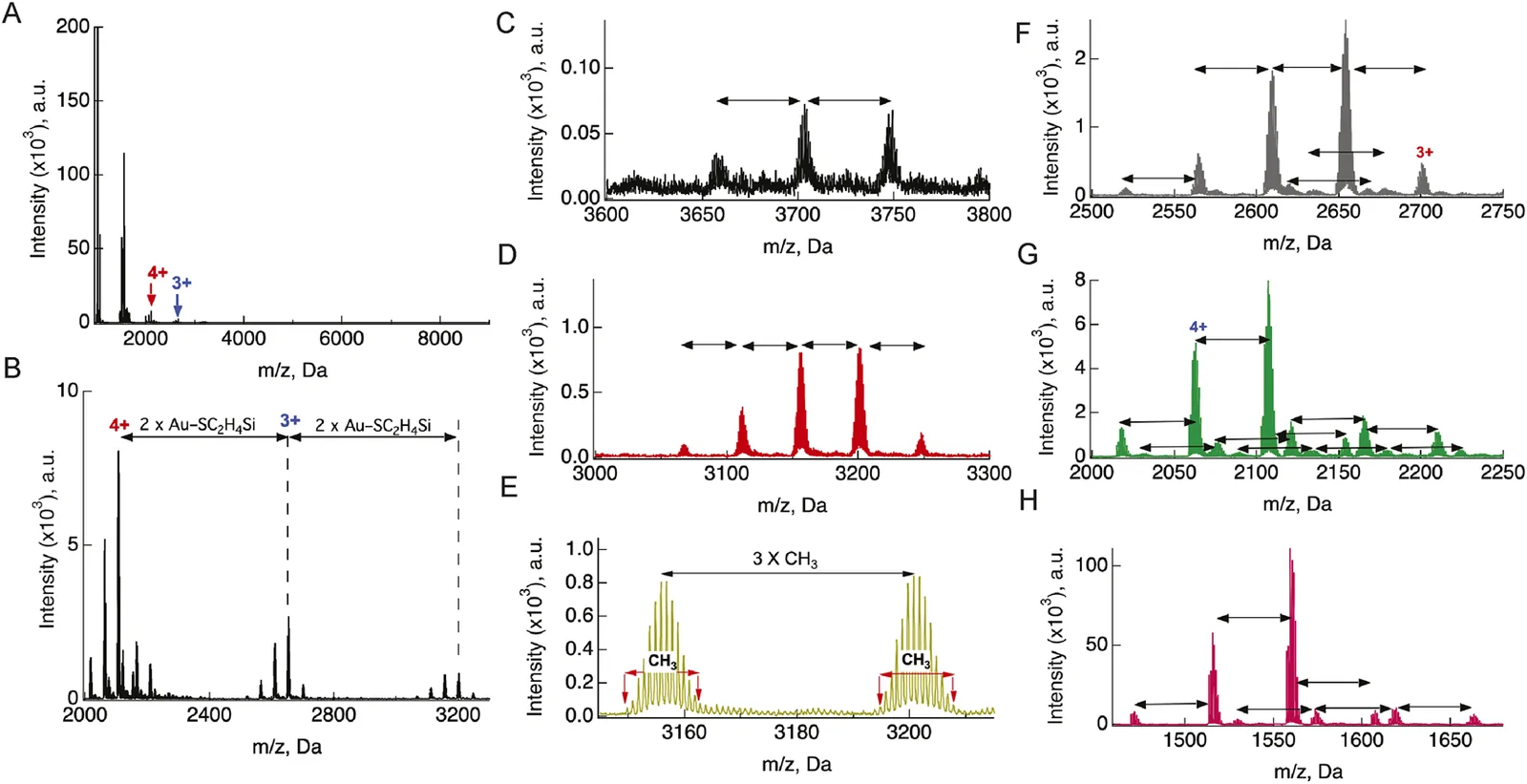
Electrospray mass spectrometry of the purified Au_25_(SC_2_H_4_Si(OCH_3_)_3_)_18_ in THF solution. **(A)** A full-range spectrum, where two arrows indicate 3+ and 4+ charged fragments. **(B)** The expanded m/z region of 2,000–3,300 Da. **(C,D)** Expanded mass region between 3,600 and 3,800 Da and 3,000–3,300 Da. **(E)** High-resolution isotopic pattern of two adjacent–CH_3_ groups and a loss of 3× CH_3_ (*ca*. 45 Da). **(F,G)** The expanded mass ranges including (3+) and (4+), and multiple–CH_3_ losses, respectively. **(H)** Lower mass range. Each double-headed arrow represents 3×CH_3_.

### 3.1 Characterization of the assembled nanostructure

Further investigation of the mass spectrometry of Au_25_(SC_2_H_4_Si(OCH_3_)_3_)_18_.TOA under different conditions, for example, captures the parent peak corresponding to the molecular mass of the entire nanocluster. Such mass spectrometry analysis would not be feasible for solid-state Au_25_-SiO_2_-particle and/or Au_25_-SiO_2_-film samples. Thus, we investigated the solid-state samples employing different analytical techniques.

Figure 3 displays X-ray photoelectron spectroscopy (XPS) analysis of the Au_25_-SiO_2_-particle. Figure 3A shows the overall XPS survey, in which several peaks corresponding to Au (4d and 4f), Si (2p), S (2s and 2p), C (1s), N (1s), and O (1s and KLL) were identified. The observed peaks support the elemental composition of Au_25_(SC_2_H_4_Si(OCH_3_)_3_)_18_.TOA. Figure 3B shows high-resolution XPS spectra of C, with peaks at 286.10 and 284.80 eV corresponding to C-OH, C-O-C, C-C, and C-H (dominant peak). The fine peak positions of Au 4f 7/2 and Si 2p (related to the presence of silane, Silicone, or Siloxane) were measured at 84.31 and 102.27 eV (Figures 3C,D). Peaks also confirmed the presence of sulfur at 168–50–161.64 eV, attributed to C-S-C and sulfide/sulfite components. These species may be produced owing to the presence of residual thiol embedded in the formed particles. Similarly, different species containing S3/2 and S1/2 have been detected, including sulfite, C-S-C, and sulfide, at energies such as 168.50, 164.32, and 162.82 eV (Figure 3E).

**FIGURE 3:**
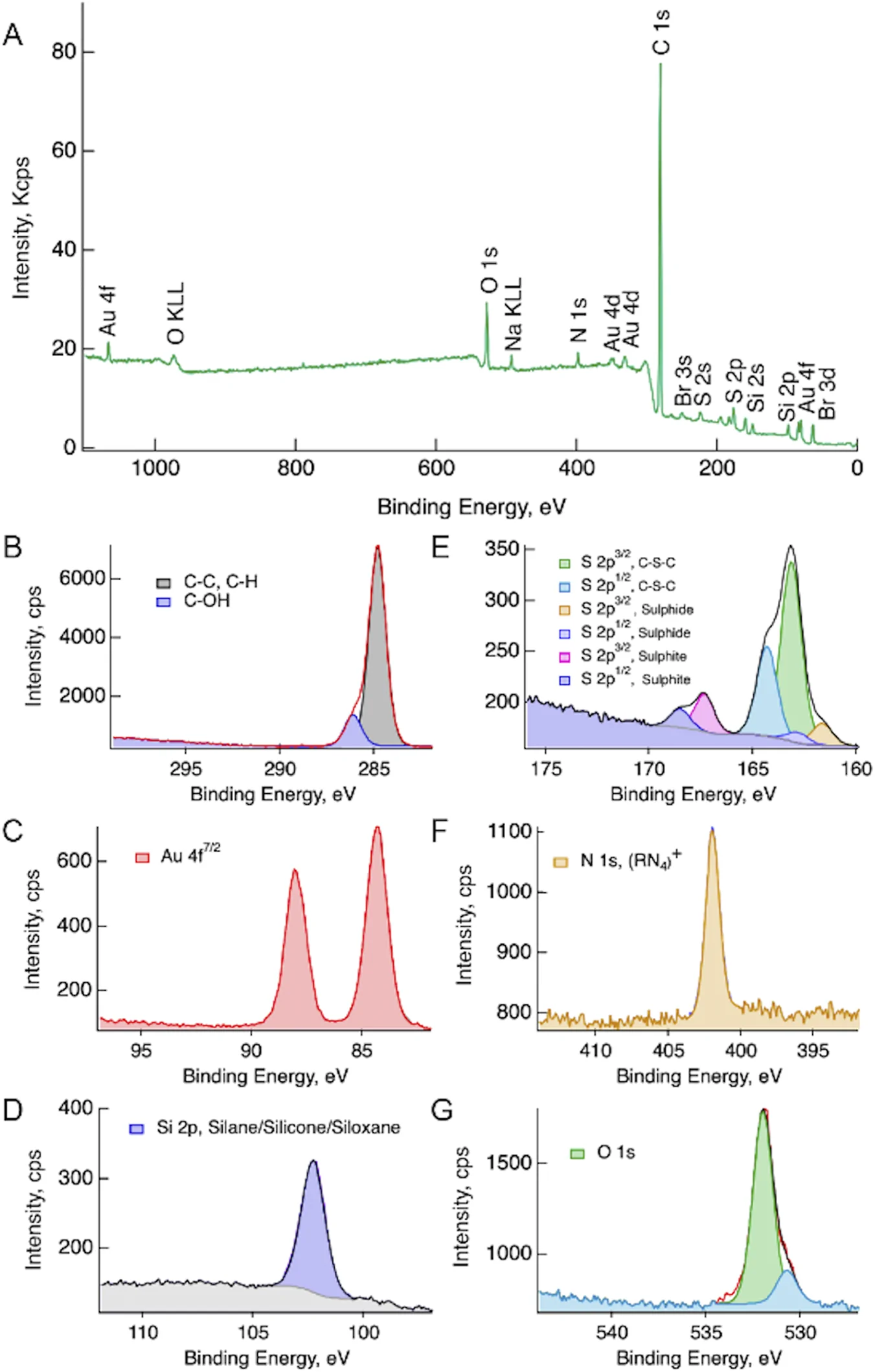
X-ray photoelectron spectroscopy of Au_25_-SiO_2_-film. **(A)** A survey spectrum. **(B**–**G)** different elemental spectra and corresponding fitting components as indicated in the insets.

Interestingly, the N 1s related to the ammonium counterion (RN_4_)^+^ was detected at 401.96 eV, supporting the negative charge state of the Au_25_(SC_2_H_4_Si(OCH_3_)_3_)_18_ (Figure 3F). The presence of Oxygen was verified with peaks at 531.97 and 530.70 eV originating from elemental oxygen in different environments within the Au_25_-SiO_2_-particle, such as Si-O or SiO_2_.

An identical XPS survey was obtained for the Au_25_-SiO_2_ film (Supplementary Figure S3), further confirming the similarity in elemental composition between the two nanostructures, i.e., the particle and the film. Scanning electron microscopy with energy-dispersive X-ray spectroscopy (SEM-EDX) was also employed to characterize the morphology and composition of both solid-state samples. The EDX elemental analysis confirmed the composition of both Au_25-_SiO_2_-particle and Au_25_-SiO_2_-film. Figure 4 represents the EDX plot and SEM image (inset) of the Au_25_-SiO_2_-particle. The presence of carbon (Kα = 0.2774), nitrogen (Kα = 0.3924), oxygen (Kα = 0.5249), silicon (Kα = 1.7398), gold (Kα = 2.1205), and sulfur (Kα = 2.3075). It can be observed that the nitrogen peak is recognized due to the presence of the tetra-*n*-methylammonium counter ion.

**FIGURE 4:**
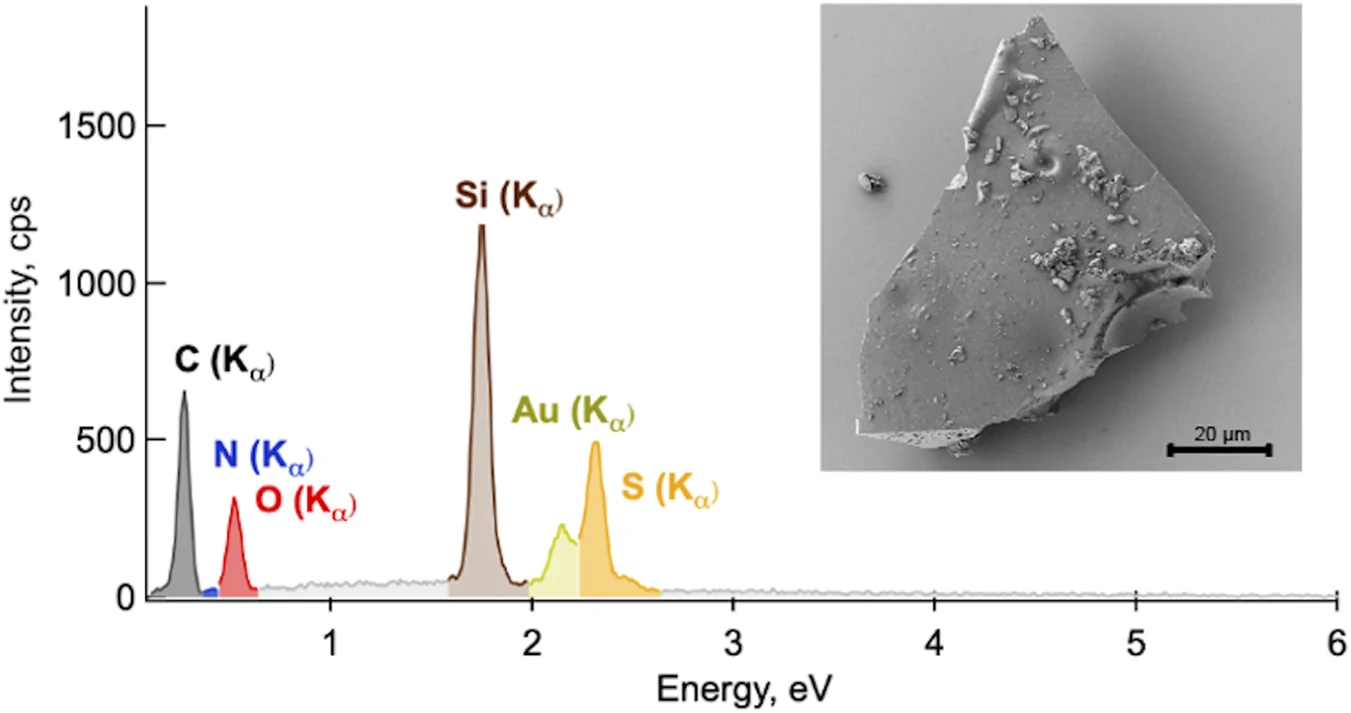
Scanning electron microscopy with energy-dispersive X-ray spectroscopy (SEM-EDX) of an Au_25_-SiO_2_-particle. The inset shows an SEM image of a sample particle.

Supplementary Figures S4A,B show wide-field and zoomed-in SEM images of the bulk, and Supplementary Figure S4C shows an SEM image of an individual Au_25_-SiO_2_-particle with a size of 98.04 ✕165.42 µm. The average *x* and *y* dimensions of 50 individual particles turn out to be 62.0 ± 3.8 µm and 48.9 ±± 0.84 µm, respectively (Supplementary Figure S4C). Supplementary Figure S5 shows a piece of Au_25_-SiO_2_-film (Supplementary Figure S5A) and a zoomed-in edge (Supplementary Figure S5B), where thickness measurements revealed a film thickness of 12.76 ± 0.036 nm. We explored the elemental distribution of Au_25_ in the SiO_2_ film (Supplementary Figure S6A) and the particle (Supplementary Figure S6B). There was an equal distribution of gold, silicon, and sulfur across both samples, indicating a homogeneous elemental pattern. The particle size and film thickness were estimated from SEM images.

X-ray powder diffraction (XRD) spectra of solid-state Au_25_-SiO_2_-particle and Au_25_-SiO_2_-film were also collected to determine the samples’ crystallinity. Supplementary Figure S7 displays the XRD profile of Au_25_-SiO_2_-particle (Supplementary Figure S7A) and Au_25_-SiO_2_-film (Supplementary Figure S7B) recorded from 3° to 80°. Each spectrum was curve-fitted using the Gaussian function. The results show a peak at 24.1° ± 0.5° (peak width: 3.12° ± 0.09°) and a broad peak at 39.4° ± 0.2° (peak width: 13.5° ± 1.0°) (Supplementary Figure S7A). A similar trace was also observed for Au_25-_SiO_2_-particle (Supplementary Figure S7B), with a peak at 24.7° ± 0.1° (peak width: 3.47° ± 0.11°) is an indication of SiO2 texture within the sol-gel material (Dubey et al., 2015). The broad peak at 40.2° ± 0.2° (peak width: 13.2° ± 0.8°) is consistent with features for Au_25_ clusters with compact *fcc* geometry published previously (Cleveland et al., 1997; Schaaff et al., 1998). The peak breadth could be an indication of the nanostructure’s small domain-size crystallinity and/or structural disorders. Further spectroscopic characterization was performed by measuring the photoluminescence (PL) spectrum of the Au_25_-SiO_2_-film sample. A portion of the thin film was excited at 532 nm, and the PL spectrum was recorded using a BR-DD charge-coupled device (CCD) camera. A cut-off filter was employed to eliminate the laser signal, and the spectrum was accumulated for 20 s.

Supplementary Figure S8 shows the obtained PL spectra of the Au_25_-SiO_2_-film (dashed line) and purified Au_25_(SC_2_H_4_Si(OCH_3_)_3_)_18_.TOA in a THF solution (dashed line). Au_25_(SC_2_H_4_Si(OCH_3_)_3_)_18_.TOA in THF matches the reported PL spectrum of Au_25_(SC_2_H_4_Ph)_18_.TOA, with an emission peak at ∼940 nm and a shoulder peak at 765 nm. One can conclude that the excitation and relaxation processes in both samples are similar and originate from *sp*-*sp* or *sp*-*d* intraband transitions (Bigioni et al., 2000). This observation provides evidence of the intactness of the Au_25_ nanoclusters within the formed nanostructure (Samanta et al., 2012). The partial PL enhancement may be due to energy transfer within and between individual embedded Au_25_ nanoclusters.

### 3.2 Catalytic performance

The catalytic performance of the Au_25_-SiO_2_ film and particle was evaluated in a typical hydrogenation reaction of 4-nitrophenol to 4-aminophenol in the presence of Au_25_-SiO_2_ materials and NaBH_4_, as a well-known and fast reaction (Scheme 1) (Zhu et al., 2014). In addition, recently, Ramaraj and colleagues reported that mono-, bi-, and alloy metallic gold and/or silver nanoshell-coated gold nanocubes (Sundarapandi et al., 2018), octahedral (Sundarapandi et al., 2019), and spherical (Sundarapandi et al., 2023) can be used as effective catalysts for nitroaromatic reduction.

The reaction progress was monitored at the 400 nm absorption peak at regular time intervals, e.g., 3 min, using UV-Visible spectroscopy (Supplementary Figure S9). The absorption peak at 400 nm corresponds to the presence of the 4-nitrophenol compound, which disappears during the reaction, and the peak at 312 nm represents the formation of 4-aminophenol. The conversion percentage of 4-nitrophenol to 4-aminophenol after 15 min of reaction turns out to be 81% and 92% for Au_25_-SiO_2_-particle and Au_25_-SiO_2_-film nanomaterials, respectively. Figure 5 shows ln C/C0, where C and C0 are the 4-nitrophenol concentrations at times t and 0, respectively.

**FIGURE 5:**
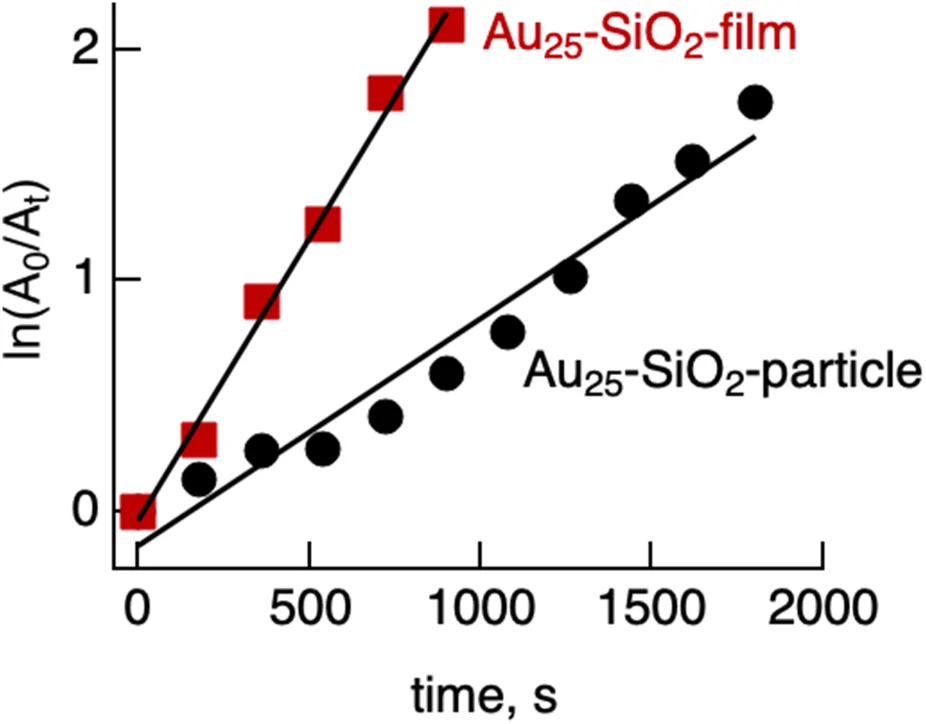
Catalytic conversion of 4-nitrophenol to 4-aminophenol using Au_25_-SiO_2_-film (rectangle) and Au_25_-SiO_2_-particle (circle). Both reactions fit a linear line.

The observed rate constant (*k*) for the catalyst was 2.44 × 10^−3^ ± 1.09 × 10^−4^ s^-1^ and 9.83 × 10^−4^ ± 6.76 × 10^−5^ s^-1^ for Au_25_-SiO_2_-film and Au_25_-SiO_2_-particle, respectively, as calculated directly from the slope of the straight line shown in Figure 5. The obtained rates are in close agreement with the reported values for silica-uncoated water-soluble (Yamamoto et al., 2012) and organic-soluble gold (Zhao et al., 2016) and silver-copper (Yuan et al., 2022) nanoclusters. The Au_25_-SiO_2_ rate is lower than that of Au_25_(SCH_2_CH_2_Ph)_18_^–^; however, the silica coating facilitates catalyst collection and reuse.

It is worth highlighting that the *k* value for Au_25_-SiO_2_-film is ∼2.5-fold higher than that of Au_25_-SiO_2_-particle. This could be attributed to the thinner SiO_2_ layer surrounding the Au_25_ nanoclusters, which provides more effective access to the reactant at the gold nanoclusters’ surface and serves as a more effective catalyst material. Indeed, this supports the crucial role of silica content in creating an efficient network, rather than in preventing site accessibility for catalysis. Table 1 summarizes the reported reaction rates of different nanomaterials. We evaluated the recycling recovery of both materials, and a 96% recovery over ten cycles was achieved. Supplementary Figure S10 displays the SEM image of Au_25_-SiO_2_-film and Au_25_-SiO_2_-particle after the catalytic reaction.

**TABLE 1:** Exemplary systems used for the p-nitrophenol to 4-aminophenol reaction.

Nanomaterial	Condition	Rate, s-1	Ref.
Au_25_(SG)_18_/DMF modified	RT, NaBH_4_	8 × 10^−3^	Yamamoto et al. (2012)
Au_24_(SCH_2_PhtBu)_20_	RT, NaBH_4_	9 × 10^−3^	Zhao et al. (2016)
Au_25_(SCH_2_CH_2_Ph)_18_^–^	RT, NaBH_4_	24.2 × 10^−3^	Zhao et al. (2016)
Au_23_(S-*c*-C_6_H11)_16_^–^	RT, NaBH_4_	37 × 10^−3^	Zhao et al. (2016)
Ag_25_Cu_4_Cl_6_H_8_(dppb)_6_ (PhC ≡C)_12_(SO_3_CF_3_)_3_	RT, NaBH_4_	1.109 × 10^−4^	Yuan et al. (2022)
Au_25_-SiO_2_-film	RT, NaBH_4_	2.44 × 10^−3^	This work
Au_25_-SiO_2_-particle	RT, NaBH_4_	9.83 × 10^−4^	This work

## 4 Conclusion

In summary, Au_25_(SC_2_H_4_Si(OCH_3_)_3_)_18_.TOA nanocluster was successfully prepared via a one-pot synthesis. The resulting nanocluster was converted into SiO_2_-embedded forms, such as particles or films, during purification. The Au_25_(SC_2_H_4_Si(OCH_3_)_3_)_18_.TOA nanocluster was characterized in solution and solid-state by employing various analytical techniques such as ESI mass spectrometry, SEM-EDX, XPS, UV-visible, and PL spectrometry to confirm the physicochemical properties and integrity of the origin of the Au_25_ nanocluster. In addition, synchrotron spectroscopy may provide some insights into both the structure and performance of the proposed morphologies. One may consider such strategies to assemble other atomically precise metal nanoclusters to generate superstructures. Additionally, due to the importance of atomically precise nanoclusters in catalytic processes, the obtained nanostructure of the Au_25_-SiO_2_-Particle and/or Au_25_-SiO_2_-film could be suitable for catalytic conversions. It has been observed that the morphology and tackiness of SiO_2_ play a critical role in the nanoassembled nanocluster, while keeping the inherent physicochemical properties intact. The 2.5-fold higher reaction rate of Au_25_-SiO_2_-film makes it a more suitable candidate for other catalytic reactions. In this architecture, the amount of SiO_2_ is enough to assemble the individual Au_25_ building blocks and does not substantially hinder catalytic properties.

## Data Availability

The original contributions presented in the study are included in the article/Supplementary Material, further inquiries can be directed to the corresponding author.
