# Supplementary material for: Controlled sol-gel nanoassembly of Au25(SR)18 nanoclusters for structural and functional integration
Source: Front Chem. 2026 Oct 12;14:1982602. doi: 10.3389/fchem.2026.1982602 (PMC13696417; doi:10.3389/fchem.2026.1982602)
Supplement: Supplementary file 1 [file DataSheet1.docx]

Supplementary Material for

***Controlled Sol–Gel Nanoassembly of Au₂₅(SR)₁₈ Clusters for Structural and Functional Integration***

Mahdi Hesari,* and Marysa Colvin

Department of Chemistry, State University of New York at Oswego, Oswego, New York, 13126, United States

E-mail: [mahdi.hesari@oswego.edu](mailto:mahdi.hesari@oswego.edu)

## 1. Sol-gel approaches towards the synthesis of Au_25_–SiO_2_ network

After primary separation, two different procedures were applied:

**Procedure (A)**: The solvent was rotary evaporated, and the residual oily materials settled overnight. Then, the excess thiol was washed with a methanol/water mixture, while the silica layer embedded in the Au_25_ core was formed during the washing procedure. The resulting Au_25_-SiO_2_ grains were collected and characterized using solid-state UV-visible spectroscopy and scanning electron microscopy with energy-dispersive X-ray spectroscopy (SEM-EDX).

**Procedure (B)**: The gravity-filtered solution of Au_25_(SC_2_H_4_Si(OCH_3_)_3_)_18_.TOA clusters were passed through column chromatography, which was filled with Sephadex LH-20. The Sephadex LH-20 was swelled in THF for 24 hours before packing in a glass column. Then, the slurry was packed in a glass column, in which the bed was capable of separating molecules based on their molecular weights (Buining et al., 1997). Figure S2 displays digital photos of the assembled SEC column while the nanocluster was eluted through the column with THF. Then, the purified Au_25_(SC_2_H_4_Si(OCH_3_)_3_)_18_ was analyzed using UV-visible and photoluminescence (PL) spectroscopy, and electrospray ionization (ESI) mass spectrometry. Next, the THF solution of Au_25_(SC_2_H_4_Si(OCH_3_)_3_)_18_.TOA containing residual water was rotary evaporated under a moderate rotation rate at room temperature. This approach resulted in the formation of a thin film of the Au_25_ nanoclusters embedded in a thin film of the SiO_2_ network.

**Figure S1.** UV-visible spectroscopic monitoring during synthesis of Au_25_(SC_2_H_4_Si(OCH_3_)_3_)_18_.TOA in THF. The inset shows the UV-Vis absorption spectrum after 24 h of reaction.

**Figure S2.** Digital photographs of size-exclusion chromatography (SEC) purification of Au_25_(SC_2_H_4_Si(OCH_3_)_3_)_18_.TOA.

**Figure S3.** X-ray photoelectron spectroscopy survey of Au_25_-in-film.

# 2 Scanning electron microscopy with electron-dispersive X-ray spectroscopy (SEM-EDX) of Au_25_-SiO_2_-film and Au_25_-SiO_2_-particle.

**Figure S4.** Scanning electron microscopy (SEM) images of (A) wide-field, (B) zoomed-in, and (C) exemplary Au_25_-SiO_2_-particle.

**Figure S5.** Scanning electron microscopy (SEM) image of (A) an individual Au_25_-SiO_2_-film, and (B) a zoomed-in area. The inset shows a histogram of measured thickness at the edge, indicated by a double-ended arrow.

We explore the elemental distribution among Au_25_-SiO_2_-film (Figure S6A) and Au_25_-SiO_2_-particle (Figure S6B). One may notice the equal distribution of gold, silicon, and sulfur across both samples, indicating a homogeneous elemental pattern.

**Figure S6.** Scanning electron microscopy with electron-dispersive X-ray spectroscopy (SEM-EDX) of Au_25_-SiO_2_-film and (A) Au_25_-SiO_2_-particle and (B) particle. The elemental color codes are carbon (dark gray), oxygen (green), Sulfur (red), Nitrogen (cyan), gold (yellow), and silicon (gray).

# 3 X-ray powder diffraction spectroscopy of Au_25_-SiO_2_ film and Au_25_-SiO_2_ particle.

**Figure S7.** X-ray powder diffraction spectroscopy of Au_25_(SC_2_H_4_Si(OCH_3_)_3_)_18_.TOA embedded in SiO_2_. (A) Au_25_-SiO_2_-particle, (B) Au_25_-SiO_2_-film. The solid lines represent fitted curves.

# 4 Solid-state photoluminescence of Au_25_-SiO_2_

Further characterization was performed by measuring the photoluminescence (PL) spectrum of the Au_25_-SiO_2_-film sample. Figure S8 shows the PL spectrum of a piece of the thin film excited using a 532 nm laser, and the PL spectrum was recorded on a BR-DD charge-coupled device (CCD) camera. A cut-off filter was employed to eliminate the laser signal, and the spectrum was accumulated for 20 seconds.

**Figure S8.** Photoluminescence spectroscopy of Au_25_(SC_2_H_4_Si(OCH_3_)_3_)_18_.TOA. Au_25_-SiO_2_-film (solid line) and Au_25_(SC_2_H_4_Si(OCH_3_)_3_)_18_.TOA in THF solution (dashed line).

# 5 Catalytic reduction reaction

The catalytic performance of the nanoassembled Au_25_-SiO_2_ film and particle was evaluated in a typical hydration reaction, specifically the reduction of 4-nitrophenol to 4-aminophenol, in the presence of metallic particles and NaBH_4_, a well-known fast reaction (Bigioni et al., 2000). This observation provides evidence of the intactness of the Au_25_ nanoclusters within the formed nanostructure (Samanta et al., 2012).

The Wu group previously reported the p-nitrophenol reduction reaction (Zhu et al., 2014). In a typical catalytic reaction, a 1 mM nitrophenol solution in a THF/water mixture (20/5, v/v) was prepared. Then, 5 mg of Au_25_-SiO_2_, either particle or film, was added. After dispersing the nanomaterial in the solution for several minutes, 40 equiv. of NaBH_4_, dissolved in ice-cold water, was added. Immediately after the addition of NaBH_4_, UV-Visible spectra were recorded at 3-minute intervals. Figure S9A shows the collected UV-Visible spectra for (A) Au_25_-SiO_2_-film, and Figure S9B shows the results for Au_25_-SiO_2_-particle. In the absence of Au_25_-SiO_2,_ no 4-nitrophenol reduction was observed. The observed noise in the collected absorption spectra is due to the fast hydrogenation reaction and H_2_ gas formation interference. The prepared catalysts, i.e., Au_25_-SiO_2_ film and particles, are stable over 6 months, as well as through multiple cycles. In addition, the materials stay intact after the catalytic reaction, as shown in Figure S10.

**Figure S9.** UV-Visible spectra of *p*-nitrophenol reduction in the presence of NaBH_4_ and (A) Au_25_-SiO_2_-film, and (B) Au_25_-SiO_2_-particle.

**Figure S10.** Scanning electron microscopy (SEM) image of (A) an individual Au_25_-SiO_2_-particle and (B) Au_25_-SiO_2_-film after the catalytic reaction.

# 6 Reference

Bigioni, T.P., Whetten, R.L., and Dag, O. (2000). Near-Infrared Luminescence from Small Gold Nanocrystals. *J. Phys. Chem. B* 104 (30), 6983-6986. doi: 10.1021/jp993867w.

Buining, P.A., Humbel, B.M., Philipse, A.P., and Verkleij, A.J. (1997). Preparation of Functional Silane-Stabilized Gold Colloids in the (Sub)nanometer Size Range. *Langmuir* 13 (15), 3921-3926. doi: 10.1021/la962071a.

Cleveland, C.L., Landman, U., Shafigullin, M.N., Stephens, P.W., and Whetten, R.L. (1997). Structural Evolution of Larger Gold Clusters. *Z. Phys. D: At., Mol. Clusters* 40 (1), 503-508. doi: 10.1007/s004600050263.

Hesari, M., and Workentin, M.S. (2014). Facile synthesis of Au_23_(SC(CH_3_)_3_)_16_ clusters. *J. Mater. Chem. C* 2 (18), 3631-3638,10.1039/C4TC00059E. doi: 10.1039/c4tc00059e.

Negishi, Y., Kurashige, W., and Kamimura, U. (2011). Isolation and Structural Characterization of an Octaneselenolate-Protected Au_25_ Cluster. *Langmuir* 27 (20), 12289-12292. doi: 10.1021/la203301p.

Samanta, A., Dhar, B.B., and Devi, R.N. (2012). Ultrasmall Gold Cluster Arrays Encapsulated in Silica Nanospheres: Applications in Fluorescence Imaging and Catalysis. *J. Phys. Chem. C* 116 (2), 1748-1754. doi: 10.1021/jp2093737.

Schaaff, T.G., Knight, G., Shafigullin, M.N., Borkman, R.F., and Whetten, R.L. (1998). Isolation and Selected Properties of a 10.4 kDa Gold:Glutathione Cluster Compound. *J. Phys. Chem. B* 102 (52), 10643-10646. doi: 10.1021/jp9830528.

Zhu, M., Zhou, S., Yao, C., Liao, L., and Wu, Z. (2014). Reduction-resistant and reduction-catalytic double-crown nickel nanoclusters. *Nanoscale* 6, 14195-14199,10.1039/C4NR04981K. doi: 10.1039/C4NR04981K.
